# Generalizing location-centric variations to enhance contactless human activity recognition

**DOI:** 10.3389/fncom.2025.1612928

**Published:** 2025-06-19

**Authors:** Fawad Khan, Syed Yaseen Shah, Jawad Ahmad, Alanoud Al Mazroa, Adnan Zahid, Muhammed Ilyas, Qammer Hussain Abbasi, Syed Aziz Shah

**Affiliations:** ^1^Research Centre for Intelligent Healthcare, Coventry University, Coventry, United Kingdom; ^2^School of Computing, Engineering and Built Environment, Glasgow Caledonian University, Glasgow, United Kingdom; ^3^Cybersecurity Center, Prince Mohammad Bin Fahd University, Alkhobar, Saudi Arabia; ^4^Department of Information Systems, College of Computer and Information Sciences, Princess Nourah bint Abdulrahman University, Riyadh, Saudi Arabia; ^5^School of Engineering and Physical Sciences Earl, Heriot-Watt University, Edinburgh, United Kingdom; ^6^College of Engineering, Department of Cybersecurity, Al Ain University, Abu Dhabi, United Arab Emirates; ^7^School of Engineering, James Watt Building (South), University of Glasgow, Glasgow, United Kingdom

**Keywords:** federated learning, human activity recognition, non-independent and identically distributed (non-IID) data, localization, weighted averaging

## Abstract

Contactless Human Activity Recognition (HAR) has played a critical role in smart healthcare and elderly care homes to monitor patient behavior, detect falls or abnormal activities in real time. The effectiveness of non-invasive HAR is often hindered by location-centric variations in Channel State Information (CSI). These variations limit the ability of HAR models to generalize across new unseen cross-domain environments, for instance, a model trained in one location might not perform well in another physical location. To address this challenge, in this study, we present a novel federated learning (FL) algorithm designed to train a robust global model from local datasets in different localizations. The proposed Federated Weighted Averaging for HAR (Fed-WAHAR) algorithm mitigates location-induced disparities, including heterogeneity and non-Independent and Identically Distributed (non-IID) data distributions. Fed-WAHAR employs a dynamic weighting approach based on local models' accuracy to improve global model classification accuracy and reduce convergence time effectively. We evaluated the performance of Fed-WAHAR using various metrics, including accuracy, precision, recall, F1 score, confusion matrix, and convergence analysis. Experimental results demonstrate that Fed-WAHAR achieves an accuracy of 85% in recognizing human activities across different locations, enhancing the ability of model to infer across new unseen locations.

## 1 Introduction

With the advancement of technologies, smart healthcare systems have enhanced the efficiency, accuracy, and accessibility of medical services to support proactive, personalized, and data-driven patient care. One such cornerstone of smart healthcare is Human Activity Recognition (HAR), particularly for settings such as elderly care homes where continuous, unobtrusive monitoring is essential. Traditionally, biosensing technologies such as electroencephalography (EEG) and electromyography (EMG) (Lévi-Strauss et al., 2025) have been used to capture detailed neurophysiological signals, aiding in the understanding neurological conditions. However, these methods often require direct physical contact and can be impractical in ambient, long-term care environments. Recent advancements in contactless wireless sensing (Kouhalvandi and Karamzadeh, 2025) especially those leveraging Channel State Information (CSI) offer a promising alternative. CSI-based HAR captures variations in wireless signal propagation caused by human motion, enabling the detection of activity patterns linked to neurological behaviors, such as tremors or gait changes (López-Delgado et al., 2025), and vital signs (Antolinos and Grajal, 2025) without compromising comfort or privacy.

This shift toward contactless HAR monitoring aligns with broader developments in localization technologies, which aim to determine the position and movement of individuals within a space. Localization can be performed effectively in an outdoor environment using the Global Position System (GPS) (Li et al., 2014). In contrast, it can be detected in an indoor environment (Saeed et al., 2023) by employing technologies such as Ultra Wide-Band (UWB), WiFi, Radio Frequency Identification (RFID), Bluetooth, cameras, and inertial sensors (Liu et al., 2017; Mendoza-Silva et al., 2019; Sophia et al., 2021; Yang et al., 2015). In addition to localization, activity recognition, for instance, sitting, standing, and walking, can be detected by inertial wearable sensors, cameras, and contactless WiFi systems. Wearable device-based localization and activity recognition pose no privacy concerns; however, camera-based has certain privacy issues, and in some countries, video surveillance is considered illegal (Klonovs et al., 2015). Contactless RF-based Human Activity Recognition (HAR) and localization approaches pose no privacy concerns and do not require the gadgets to be worn as wearable sensors.

Indoor localization techniques are helpful and significant in various domains, such as disaster prediction and recovery, healthcare, intelligent transportation, and navigation. In healthcare, both localization and activity-tracking techniques are desirable and essential to identify critical activities, such as falls in elderly patients due to dementia. Due to advancements in healthcare diagnostics, the elderly population is growing with increasing life expectancy. According to one of the statistical figures shared by the United Nations (UN), by 2050 elderly population is projected to be 2.1 billion (Naja et al., 2017). This rise in elderly population has rapidly dwindled hospitals' capacity (Haider et al., 2019).

Radio Frequency (RF) based human activity recognition and localization has been researched recently (Khan et al., 2025; Yurtman and Barshan, 2016; Bibbò et al., 2022; Chen et al., 2021) due to its privacy-preserving, non-invasive, and contactless nature. Indoor localization is affected by several factors, including the noise level, signal attenuation, and the type of surroundings (for instance, tables and chairs) where the activities are performed. Different participants performing activities, the location of the RF transmitter/receiver, and the exact location of the performed activity in an indoor environment affect localization and HAR.

RF-based activity recognition can be realized by systems employing the Channel State Information (CSI) or the Received Signal Strength Indicator (RSSI). As stated by Yang et al. (2013), CSI is fine-grained, but RSSI provides coarse information. Another study demonstrated that CSI can be employed to localize, detect, and distinguish human activities by examining the amplitudes of RF signal upon occurrence of a human activity (Chopra et al., 2016). Several existing works have utilized the CSI of RF signals similar to Wi-Fi for detecting activities such as vital signs Wang et al. (2020), body motions (Taylor et al., 2020), and localization and tracking (Shi et al., 2018).

In addition to the numerous advantages of CSI for localization and activity monitoring, one significant challenge is its limited ability to perform well in new, unseen cross-domain locations. For instance, an optimized HAR model for one environment might not perform well in another. The generation of the dataset and training of the HAR model involves Sensors, Participants, Environmental Conditions, and Signal Processing (SPECS). Even if only the environmental conditions were changed, for instance, training in one location and inference in another, the model performance for HAR could be affected due to location disparities. An obvious solution to this problem is to collect samples from new environments and optimize the model to enhance the recognition accuracy. However, this approach is too tedious and repetitive.

Federated learning (FL) (Ma et al., 2021; Hong et al., 2022) is one such technique, which can incorporate location-centric characteristics and features from multiple clients in different locations for training the global model. The model can then infer better in new locations/places without requiring extensive data recording, annotations, and model training for each new location. In addition, FL allows collaborative model training among clients without sharing the critical data or dataset, addressing privacy and ethical considerations.

In addition to the numerous advantages of FL, there are associated challenges in FL when the client's datasets are non-homogeneous and non-Independent and Identically Distributed (non-IID). The heterogeneous and non-IID characteristics in HAR exist due to SPECS. Moreover, the sampling and label distribution across domains can lead to heterogeneity. This can affect the global model's convergence as the local models drift according to their data distribution and class labels. This leads to delays in global model convergence, lower accuracy, and an inability to generalize across new locations, with enhanced computation and communications costs.

**Figure 1:**
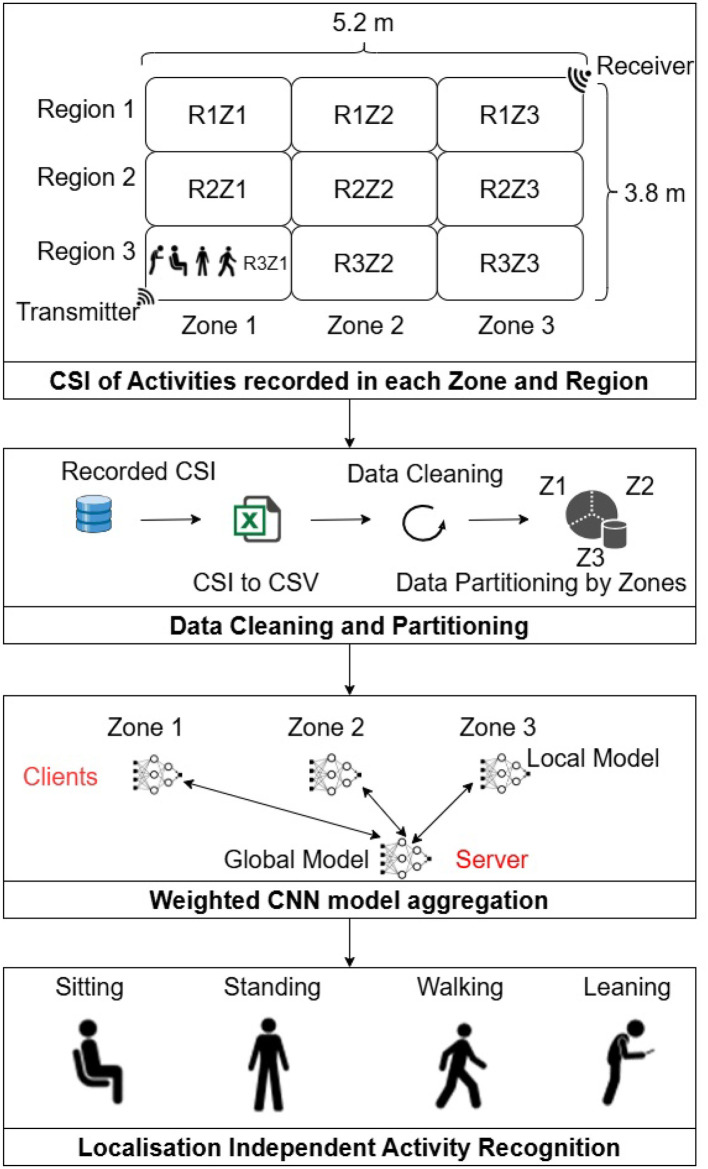
Flow diagram of RF-enabled setup for location independent human activity recognition.

In this study, we employ RF-based Wi-Fi sensing for HAR, which is readily available in every home. The primary Wi-Fi device used is a software-defined radio (SDR). We enhance the trained FL global model's ability to generalize location-based differences and variations in CSI across different zones where activities were performed. The proposed Federated Weighted Averaging for HAR (Fed-WAHAR) considers the non-IID and non-homogeneity challenges in FL, leading to early convergence, enhanced accuracy, and lower computational and communication costs. Figure 1 presents the block diagram representing all steps from data collection, dataset generation, model training and aggregation, and activity recognition.

The rest of the study is organized as follows: Section 2 presents the literature review of recent works on FL-based HAR. Section 3 details the employed dataset and its characteristics, while Section 4 presents the proposed Fed-WAHAR algorithm. Section 5 provides the analysis and results, and Section 6 concludes the study.

## 2 Literature review

In this section, we review recent works on FL-based HAR considering the metrics of underlying sensing technology, employed algorithm, non-IID dataset setting among clients, localization, and employed variant of FL.

Wen et al. (2024) focused on addressing the Non-IID data challenges in HAR by the Federated Parameters Averaging (FedPA) algorithm. The proposed FedPA algorithm employed one-dimensional 1-D convolution neural network (CCN) and long short-term memory (LSTM) models and assigned varying model weights to local clients based on their contribution to enhance the efficacy of both the global and local models at clients. They employed the PAMAP2 and the RealWorld dataset based on Intertial Measuring Units (IMU) to demonstrate the results.

To minimize the training loss across several local client models, Ouyang et al. (2022) proposed a clustering-based FL (ClusterFL) approach to increase the accuracy and reduce communication overhead. They employed multiple datasets based on IMU, camera, and UWB in the IID data setting by using the CNN algorithm. In another work by Xiao et al. (2021), they employed various IMU datasets WISDM, UCI-HAR 2012, OPPORTUNITY, and PAMAP2 under the homogeneous data setting to propose an enhanced feature extraction algorithm for HAR.

Hernandez and Bulut (2021) demonstrated HAR in co-located environments using RF-WiFi sensing. For the generation of the dataset, they performed four activities: sit, stand up, stand, and sit down in a round-robin manner in bedroom, dining room, and office environment. They demonstrated that the FL model trained on various locations can infer better in new unseen locations. However, the proposed CNN algorithm based on FedAvg is for homogeneous and IID data distribution across all the clients.

Sozinov et al. (2018) compared a HAR CNN classifier in three settings: (1) non-IID data, (2) skewed data, and (3) uniform data. For the non-IID dataset, the FL clients have at most two activities from the original set of total activities, with one activity having 50% fewer data samples than the other. All the clients have equal data samples from the dataset for a uniform data setting. They employed an IMU-based HAR dataset (Stisen et al., 2015) to demonstrate that FL for HAR is robust under various settings and produces models with comparable accuracy to centralized machine learning.

Cheng et al. (2023) proposed a prototype-based HAR, where instead of aggregating the local models, the activity prototypes from the clients are shared to handle heterogeneity issues in non-IID data. Their approach reduces communication costs by sharing lightweight prototypes instead of gradients, where a prototype for an activity class is the mean of all feature vectors of that activity class in the client's local dataset. The process works by each client sending its activity prototypes to the server, which aggregates the prototypes from all clients to reduce the data disparity caused by data heterogeneity among clients. Prototype aggregation captures a more general representation of each activity as the knowledge is combined from several clients, reducing data disparity among clients.

Li et al. (2023) presented CARING, a FL framework for cross-domain Wi-Fi-based HAR. CARING introduced a collaborative learning paradigm that enabled knowledge sharing across multiple heterogeneous deployment environments. They addressed domain shift inconsistencies due to the physical appearances of domains and label imbalance across distributed clients. They introduced a noise-dispelling scheme to isolate activity features from domain noise, a hybrid CNN-RNN model for robust feature extraction, and dynamic weighting for model updates.

Zhang et al. (2025) introduced WiFed-CHAR, an FL framework for cross-environment HAR using Wi-Fi CSI. The framework addressed issues of data heterogeneity and limited training samples in new environments. They employed a cloud-edge collaboration paradigm to enable environments with similar recognition tasks to collaboratively learn and share feature extraction knowledge via a task-based HAR knowledge base. The collaborative learning pipeline consisted of whole-level optimization for feature extraction, hierarchical clustering by similarity, and partial-level optimization to generate specialized HAR modules.

Albogamy (2025) proposed an FL framework for IoMT-enhanced HAR using a hybrid LSTM-GRU architecture. The framework targeted privacy-preserving and decentralized HAR in healthcare and smart environments, where wearable sensors generate sensitive user data. The model integrated 1D convolutional layers for local pattern detection with LSTM-GRU units for capturing complex temporal dependencies and introduced an attention mechanism to emphasize critical features. To address heterogeneity and data imbalance, the framework employed weighted FedAvg.

Table 1 compares Fed-WAHAR with several existing works. In contrast to other works, Fed-WAHAR presents a novel FL algorithm that directly tackles the critical challenge of location-centric variations in CSI-based HAR, an area often underexplored in existing FL-HAR frameworks. Existing works focused on user-level personalization or inter-device heterogeneity; however, this research prioritizes real-world spatial variability, presenting an accuracy-driven aggregation strategy to improve convergence and cross-domain performance.

**Table 1:** Summary of existing HAR and FL approaches.

**References**	**Sensing technology**	**Algorithm**	**Non-IID**	**Localization**	**FL variant**
Wen et al. (2024)	IMU	CNN-LSTM	Y	N	FedPA
Ouyang et al. (2022)	IMU, Camera,	CNN	N	N	ClusterFL
	UWB				
Hernandez and Bulut (2021)	RF-WiFi	CNN	N	Y	FedAvg
Sozinov et al. (2018)	IMU	CNN	Y	N	FedAvg
Xiao et al. (2021)	IMU	CNN-LSTM	N	N	FedAvg
Cheng et al. (2023)	IMU	CNN	Y	N	Prototype guided FL Li et al. (2023)	RF-WiFi	CNN-RNN	Y	Y	Weighted FL
Zhang et al. (2025)	RF-WiFi	MobileNetV3	Y	Y	WiFed-CHAR
Albogamy (2025)	IMU	LSTM-GRU	Y	N	Weighted FL
		CNN			
Fed-WAHAR	RF-WiFi	CNN	Y	Y	Weighted FL

## 3 Dataset description

The HAR dataset is generated at the University of Glasgow, UK Khan et al. (2022). The dataset is generated in an area of 5.2 × 3.8 *m*^2^. As seen from Figure 1, the area is partitioned into three regions and three zones, and the partitions are, respectively, named according to zone and region number, for instance, R1Z1, R1Z2, and R3Z3. To capture the activities using CSI, the USRP (National Instruments X310/X300) transmitter and receiver devices were positioned in opposite corners of the room at a 45-degree angle facing each other. Sitting, standing, and leaning activities are performed in all partitions. Walking is performed in three partitions of a zone; for instance, walking in partitions R1Z3, R2Z3, and R3Z3 is attributed to one walking activity. CSI was also gathered across each partition when no activity was performed.

The signal processing pipeline involved extracting the amplitude of CSI from complex OFDM subcarrier data (64 subcarriers). It was preprocessed using GNU Radio flowgraphs and Python scripts, with configurable parameters: 3.75 GHz centered frequency and transmitter/receiver gain levels (70/50 dB). Data cleansing was conducted using Scikit-learn's SimpleImputer, appending the missing values via row-wise mean substitution.

**Figure 2:**
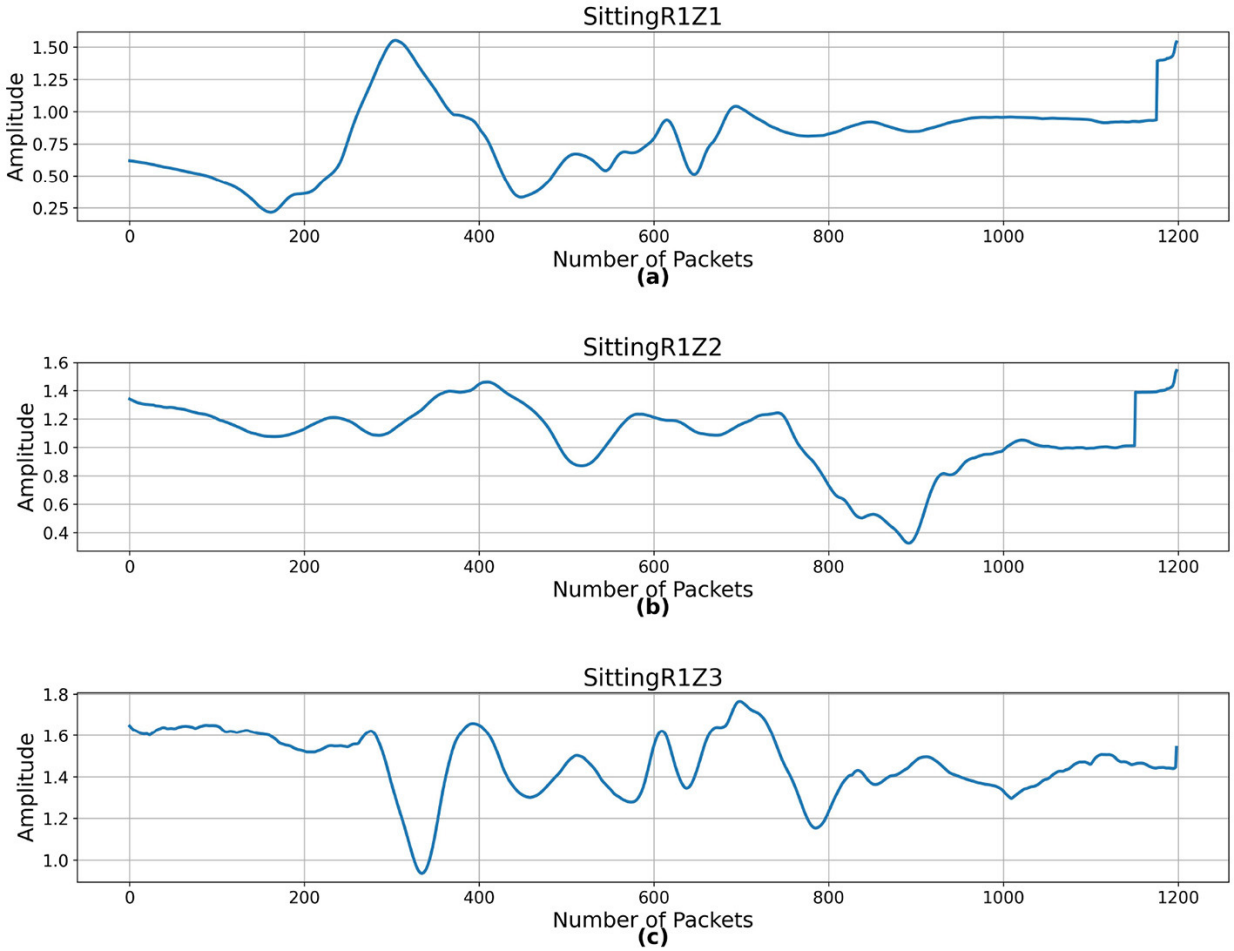
CSI samples of sitting activity across different localizations. **(a)** Region R1 and zone Z1, **(b)** region R1 and zone Z2, and **(c)** region R1 and zone Z3.

**Figure 3:**
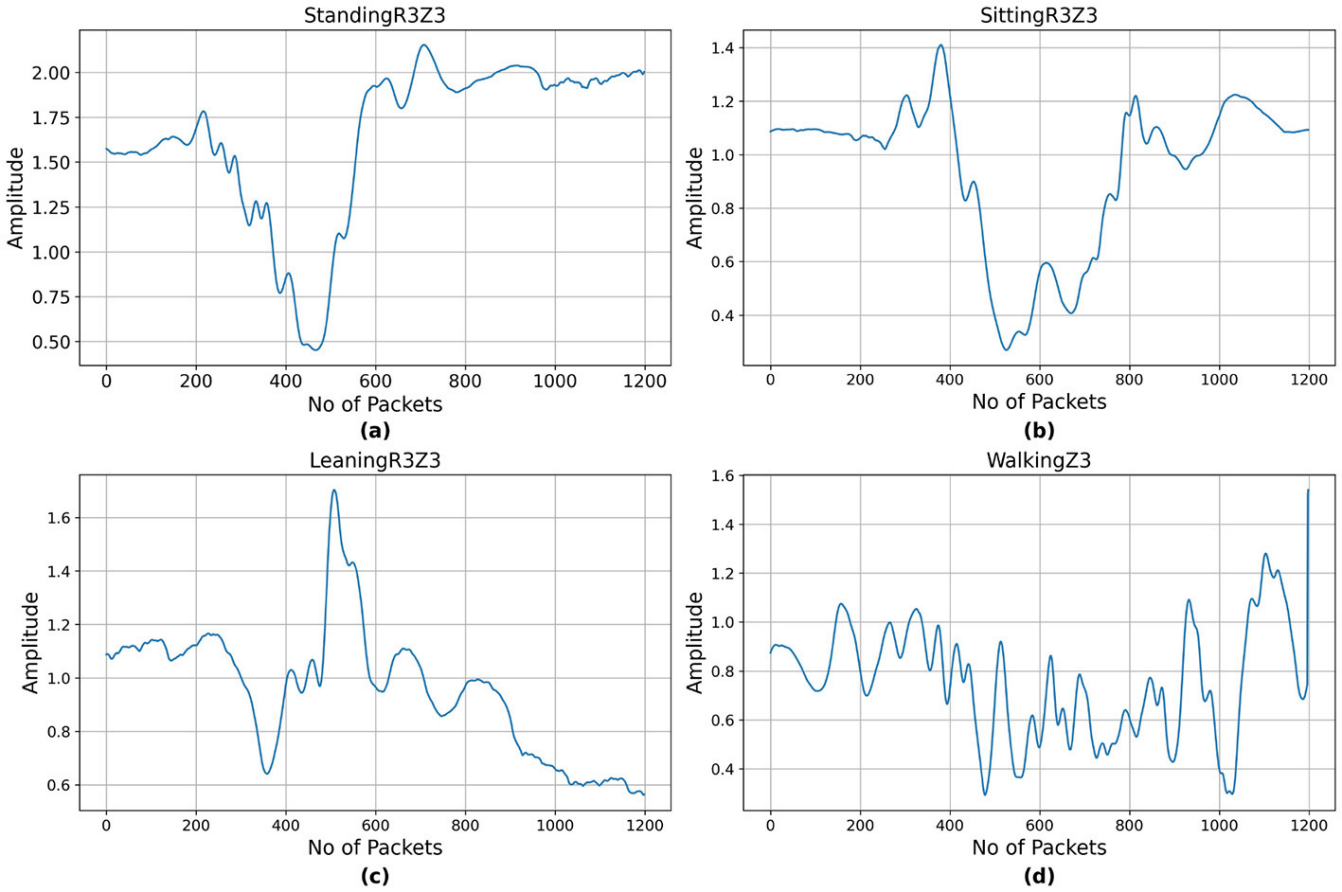
CSI samples of different activities in region R3 and zone Z3. **(a)** Standing, **(b)** sitting, **(c)** leaning, and **(d)** walking.

Figure 2 presents the CSI of sitting activity performed across region R1 and zones Z1, Z2, and Z3 localizations. As CSI is sensitive to the environment, including the location of the transmitter, receiver, and obstacles, the activities performed in different localizations have distinct signal patterns due to varying multipath effects and signal propagation paths. Figure 3 presents the CSI of standing, leaning, sitting, and walking activity across region R3 and zone Z3. CSI amplitude for static activities such as standing, sitting, and leaning remains relatively stable with minor fluctuations. For a dynamic activity such as walking, CSI amplitudes exhibit higher variability as seen from Figure 3d.

**Figure 4:**
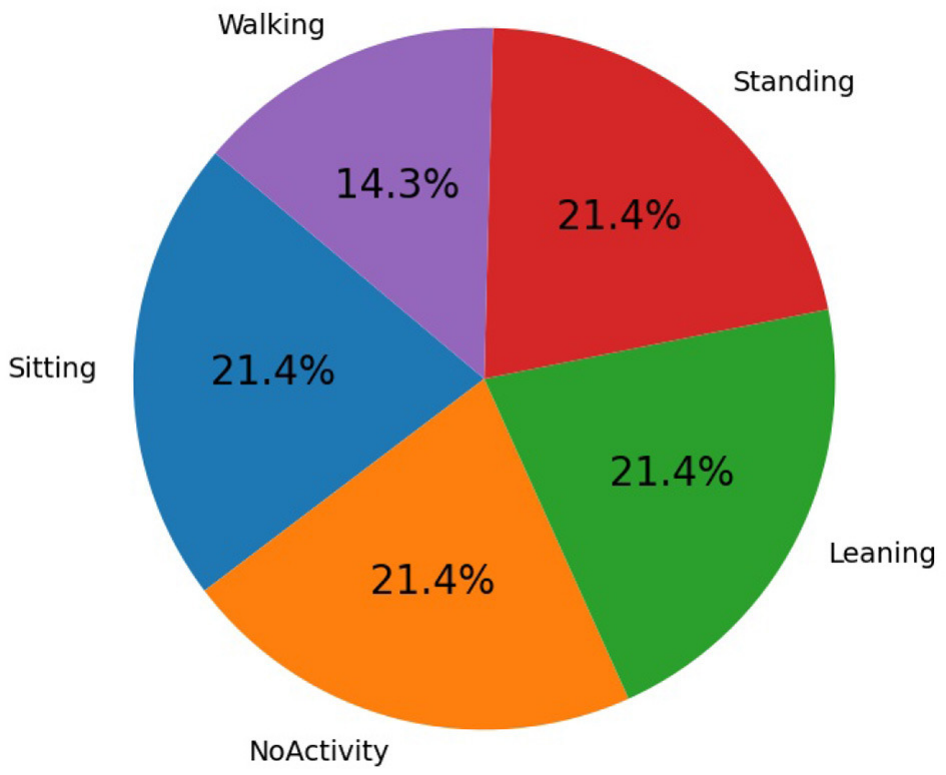
Total sample distribution across activity classes.

**Figure 5:**
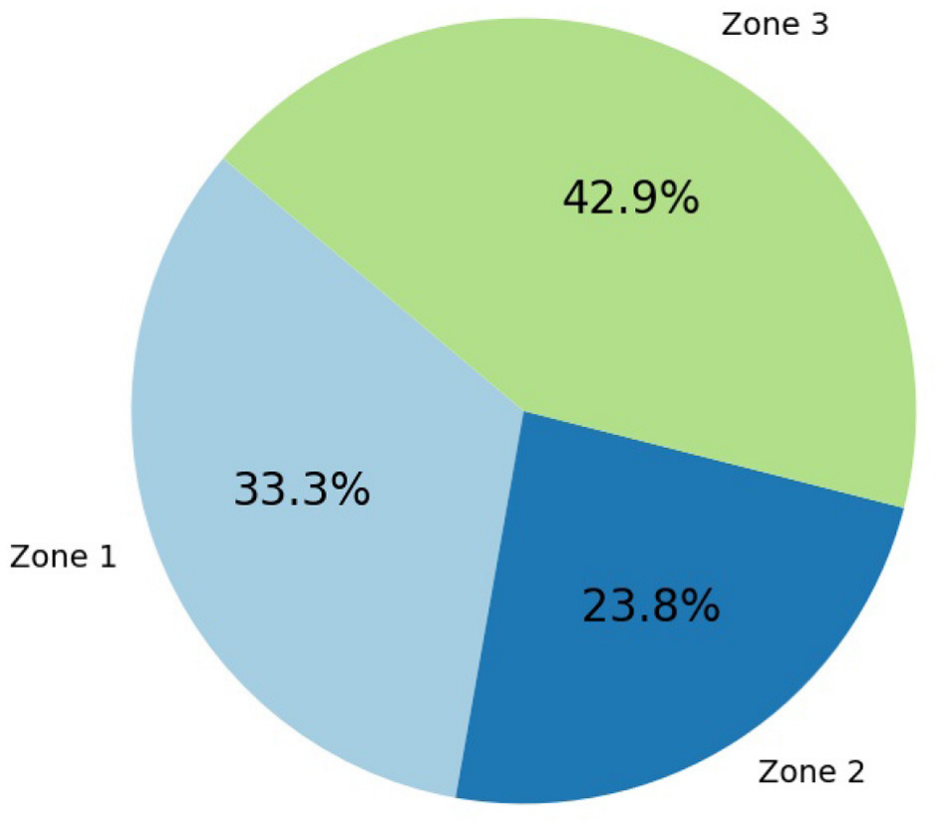
Total sample distribution across zones.

The recorded CSI from USRP was transformed into a CSV file. The dataset consists of 4,200 samples and 1,252 features. There are five activity labels: “Sitting,” “Leaning,” “Standing,” “Walking,” and “NoActivity.” Figure 4 indicates the distribution of activities, where it can be seen that the “Walking” activity has a significantly smaller number of samples than other activities, making the dataset non-IID. To realize localization and federated learning for three clients in three zones, we merged the similar activity samples from partitions in each zone; for instance, sitting activities in Zone1 and regions R1, R2, and R3 were grouped as sitting-Z1 = {sitting-R1Z1, sitting-R2Z1, sitting-R3Z1} by removing the region label specifiers. The distributions of activity samples across each zone can be seen in Figure 5 and correspond precisely to how the activities were recorded in a specific zone. As seen from Figure 5, the number of samples in Zone2 is less than others, making this setting non-homogeneous and non-IID.

The data are non-IID, as the class-level distribution and zone-level contributions to the entire dataset are imbalanced. This non-IID characteristic is typical in federated learning scenarios, where client datasets (across cross-domains or zones) have heterogeneous distributions. This affects the global model's convergence and performance across zones, as discussed in a subsequent section.

Regarding data cleaning, we further removed the zone indexes from the labels so that overall, we have five labels across all three zones, i.e., we transformed sitting-Z1 to sitting. Then, the zone datasets were concatenated for label encoding, and the datasets were again partitioned accordingly to zones. Each client dataset in the respective zone is partitioned into 80:20 training and testing samples. The testing samples are required to evaluate the accuracy of the client's local models during each round of the FL training process. These testing samples are also employed to assess the global model accuracy across different zones after the end of the training process.

## 4 Methodology

In this section, we present the proposed federated weighted averaging algorithm for HAR to cater to the heterogeneity and non-IID nature of the data across clients in zones. The proposed Fed-WAHAR algorithm generalizes the model's ability to adapt to activities detected in different zones by capturing the temporal and spatial patterns in the data. The algorithm uses the client's local model accuracy to determine its weights based on his contribution to the training process. For *j*^*th*^ client *C*_*j*_, the weight *W*_*j*_ is determined by Equation 1, where *Acc*_*j*_ is the accuracy of the locally trained model during each round of the FL process at *C*_*j*_, and *n* is the total number of clients.


(1) $$\begin{array}{l} W_{j}=\frac{Acc_{j}}{SA}\;where\;SA=\overset{n}{\underset{j=1}{\sum }}Acc_{j} \end{array}$$

These weights influence how each client's locally trained model parameters are aggregated into the global model (Ma et al., 2021; Chourasia et al., 2024). For instance, clients with larger datasets or higher local model accuracies significantly impact the global update. The algorithm prioritizes clients with higher relevance and better performance to update the global model, thereby enhancing model robustness, accelerating its convergence, and improving accuracy. The weighted update of each layer of the global model from local models is determined by Equation 2, where *l* indicates the layer of CNN model, ω_*o*_ represents the updated parameters of global model, and ω_*o, j*_ indicates the local model parameters for *j*^*th*^ local client.


(2) $$\begin{array}{l} \omega_{o}\left[l\right]\leftarrow \overset{n}{\underset{j=1}{\sum }}W_{j}\cdot \omega_{o,j}\left[l\right] \end{array}$$

### 4.1 CNN

The CNN consists of two 1D Convolution layers, two MaxPooling layers, a flattened layer, and a fully connected dense layer. The kernel size of the convolution layer is 3 with ReLU as the activation function, and the dense layer consists of 100 neurons with a softmax activation function. The layered architecture is detailed in Table 2. The CNN instance is shared with the server and the clients, as seen in Figure 1.

**Table 2:** Layered architecture of CNN model.

**Layer (type)**	**Output shape**	**Parameters**
*Conv*1*D*_1_	(None, 1249, 64)	256
*MaxPooling*1*D*_1_	(None, 624, 64)	0
*Conv*1*D*_2_	(None, 622, 128)	24,704
*MaxPooling*1*D*_2_	(None, 311, 128)	0
*Flatten*_1_	(None, 39808)	0
*Dense*_1_	(None, 100)	3,980,900
*Dense*_2_	(None, 5)	505
Total parameters: 4,006,365		
Trainable parameters: 4,006,365		
Non-trainable parameters: 0		

### 4.2 Fed-WAHAR

In this subsection, we discuss the proposed Fed-WAHAR algorithm, which takes as input the client's zone-specific training and testing datasets *C*_1_:{*Z*_1, *train*_, *Z*_1, *test*_}, *C*_2_:{*Z*_2, *train*_, *Z*_2, *test*_}, *C*_3_:{*Z*_3, *train*_, *Z*_3, *test*_} to generate the global model *G*_θ_ which will realize the localization effectively. Each client holds its training and testing dataset separately according to its zone, as these were recorded.

Initially, a global model *G*_θ_ is initialized at the server, and its parameters ω_*o*_[*l*] are shared with the clients *C*_*j*_ as in lines 1–2 of Algorithm 1, where *l* represents the layer of the CNN model. Then, for every round, each client *C*_*j*_ individually updates its local model *L*_θ, *j*_ based on the received global model parameters ω_*o*_[*l*], its local dataset *Z*_*j, train*_, epoch value *ep*, and batch size *bs* as in lines 4–6 of the algorithm. Afterwards, each client locally evaluates its trained model *L*_θ, *j*_ on its test dataset *Z*_*j, test*_ to determine the accuracy in line 7. The clients then share their respective model accuracy *Acc*_*j*_ and local model weights ω_*o, j*_[*l*] with the server, as in line 8.

**Algorithm 1:** Fed-WAHAR.

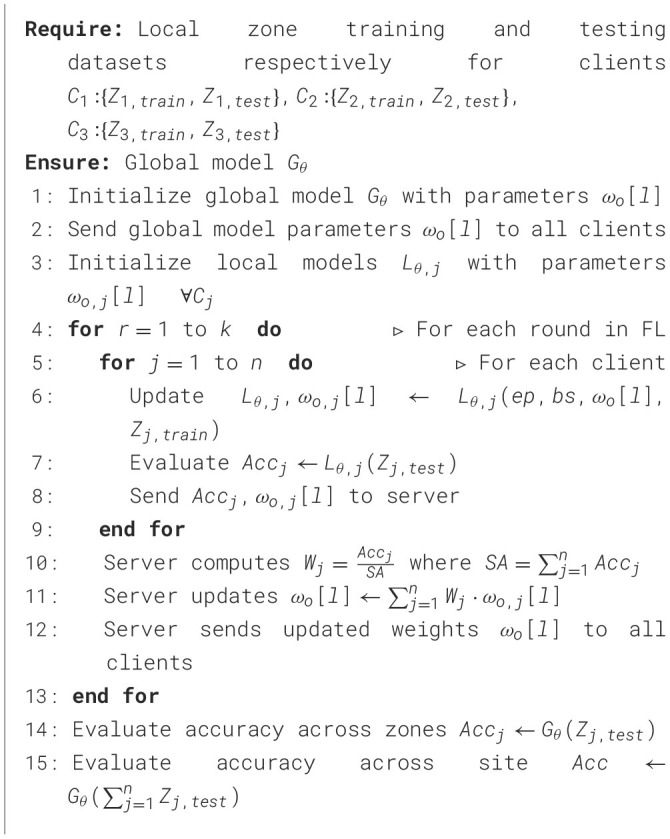

To update the global model after each FL round, the server evaluates the optimal weights *W*_*j*_ for each client upon receiving updates from each client. For that, the server first evaluates the weighted average of accuracy reported by clients so that the clients with higher accuracy can contribute more to the global model update in line 10. Thereafter, the server updates the global model based on the updated client's weight in line 11. The server finally sends the clients the updated global model weights ω_*j*_[*l*].

Once the FL training over the *r* number of rounds is completed, the performance of Fed-WAHAR is evaluated. To assess the performance of the globally trained algorithm *G*_θ_ across each zone, its accuracy across each zone is determined using the particular zone test dataset *Z*_*j, test*_ as in line 14.

## 5 Results and discussion

**Figure 6:**
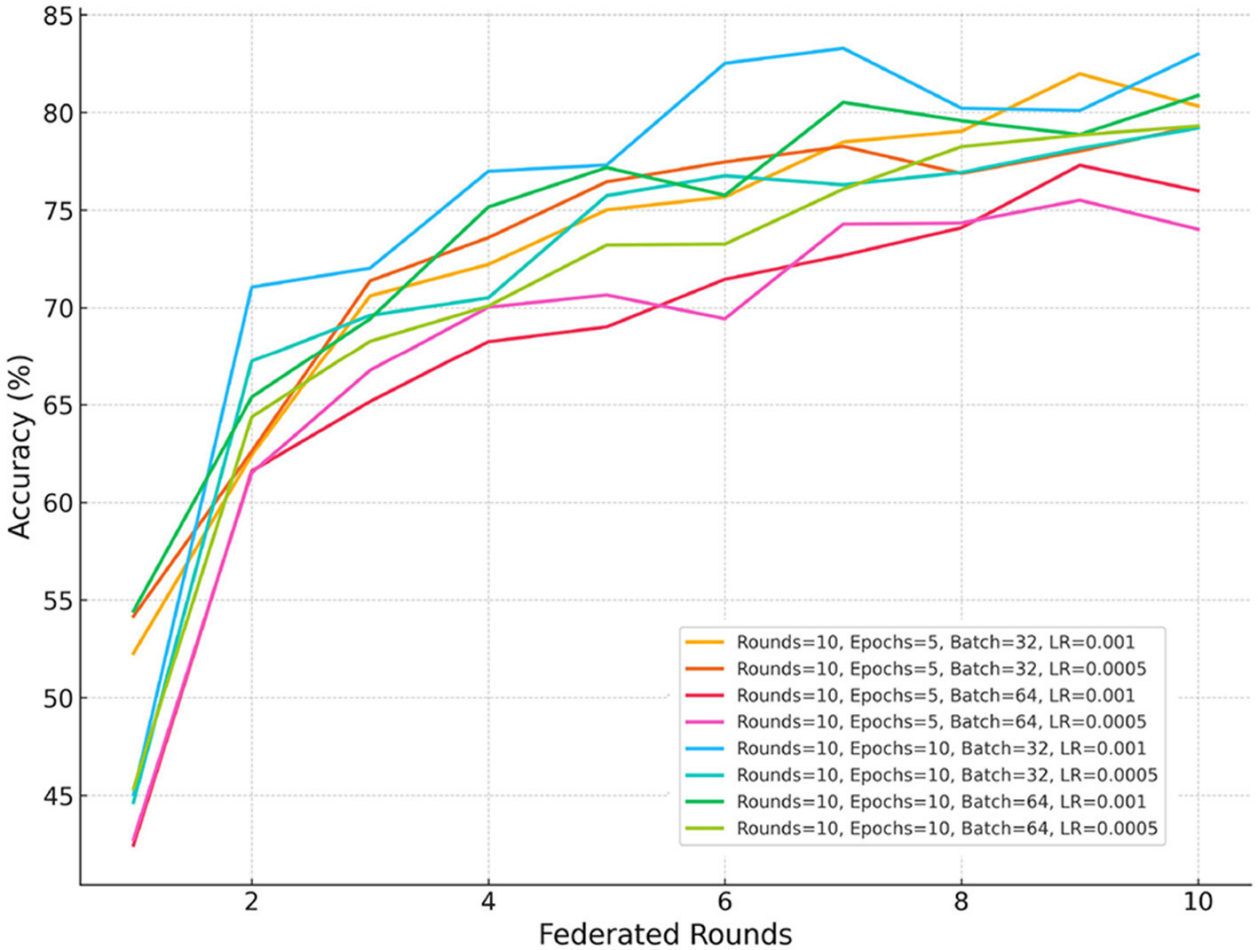
Optimal hyperparameters.

This section presents the results of the proposed Fed-WAHAR, highlighting its performance under various metrics and conventional techniques. All the experiments were performed on an AMD Ryzen 7 4800H Radeon Graphics Laptop with a processor running at 2.90 GHz and supported with 16 GB RAM. The implementation of Fed-WAHAR employed machine learning libraries, including TensorFlow and Scikit-Learn. We evaluated Fed-WAHAR against the metrics, including accuracy, precision, recall, F1 score, confusion matrix, communication cost, and convergence time. As detailed in Section 3, the dataset was systematically partitioned across zones, preserving each zone's natural data generation characteristics. Furthermore, each zone dataset is split into training (80%) and testing (20%) datasets. We conducted numerous experiments to determine the optimal hyperparameters for the training of Fed-WAHAR, including the number of epochs, batch size, and learning rate. As seen from Figure 6, a Learning Rate (LR) of 0.001 exhibited an accuracy of over 80% in 10 rounds of FL. In addition, a batch size of 32 and an epoch value of 5 proved to be optimal for ensuring early convergence of the model, striking a balance between computational efficiency and model performance.

**Figure 7:**
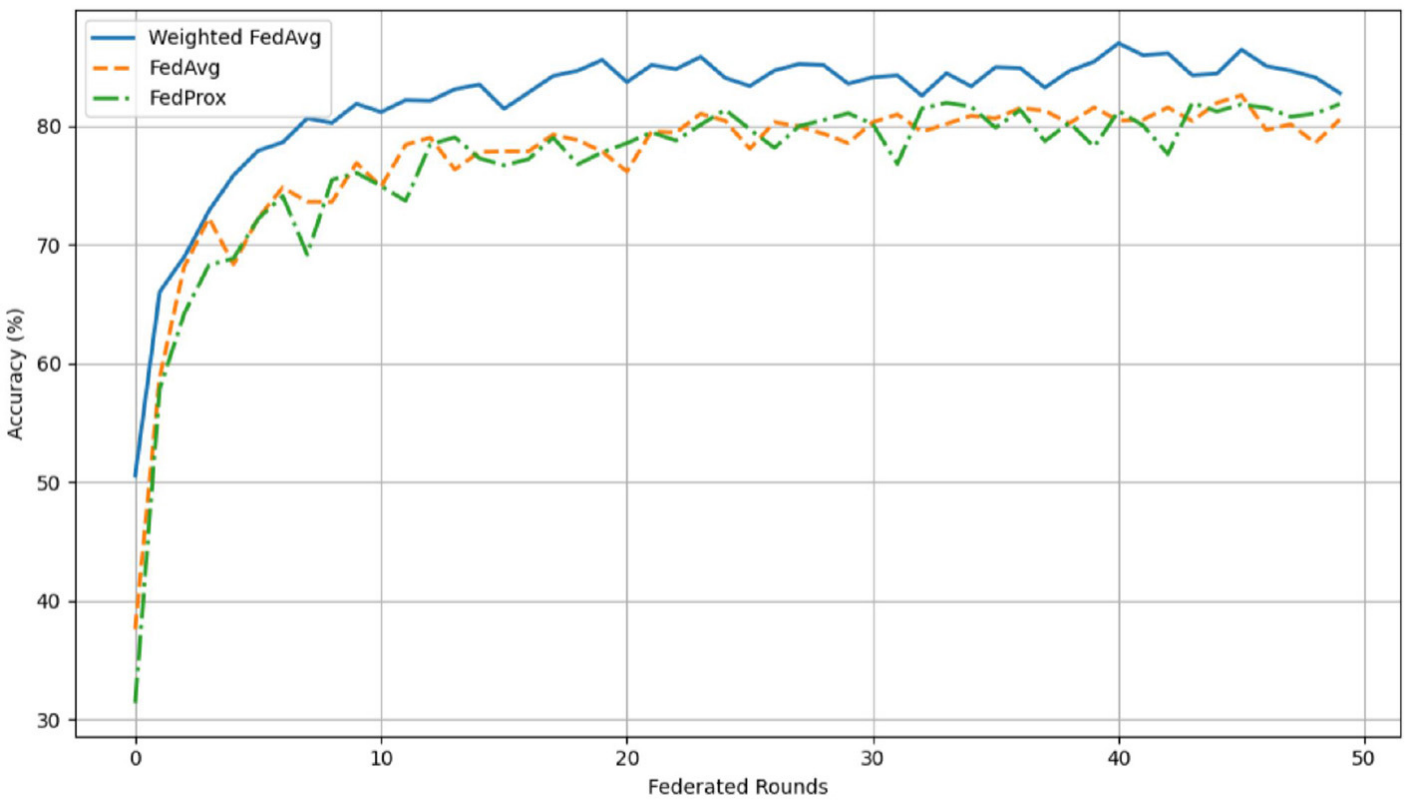
Convergence analysis.

The comparison of Fed-WAHAR with the standard Fed-Avg algorithm and FedProx Li et al. (2020) further illustrates the advantage of the proposed algorithm. As seen from Figure 7, Fed-WAHAR attained a test accuracy of 85% within just 25 rounds of training, while FedAvg and FedProx plateau ~81%–82% and require more rounds to reach comparable accuracy. The results also highlight that FedProx offers only marginal improvements over FedAvg, indicating limited gains from proximal regularization in this setting. In contrast, the accuracy-weighted aggregation of Fed-WAHAR enables faster convergence and more stable training dynamics.

This significant reduction in a number of training rounds to attain an accuracy threshold directly translates into enhanced communication efficiency, as each round involves the exchange of model parameters between the clients and the server. The total communication cost for a single round of FL between three clients and a server is 62.5 megabytes (MB). As Fed-WAHAR converges the model earlier, it improves communication efficiency by 50%.

We assessed the global model's performance on each client's local test dataset to evaluate its fairness and consistency across clients. Table 3 presents a classification report of Fed-WAHAR in different zones. The testing accuracy in Zone Z2 is 87.50%. It is the highest among all zones because, within Z2, the distribution of labels is balanced, i.e., the walking, standing, sitting, leaning, and no-activity all have the same number of samples. Z3 has the most significant samples among all zones and slightly less accuracy of 86.39%, which can be attributed to data heterogeneity and imbalanced data distribution, and the number of walking activity samples is half in contrast to other activities. Fed-WAHAR has an overall test accuracy of 85.48% over accumulated test datasets from all the zones.

**Table 3:** Classification report of Fed-WAHAR on test datasets in different zones.

**Zone**	**Precision (%)**	**Recall (%)**	**F1 (%)**	**Acc (%)**
Z1	82.99	82.86	82.74	82.86
Z2	87.61	87.50	87.51	87.50
Z3	86.61	86.39	86.26	86.39
All zones	85.61	85.48	85.44	85.48

**Figure 8:**
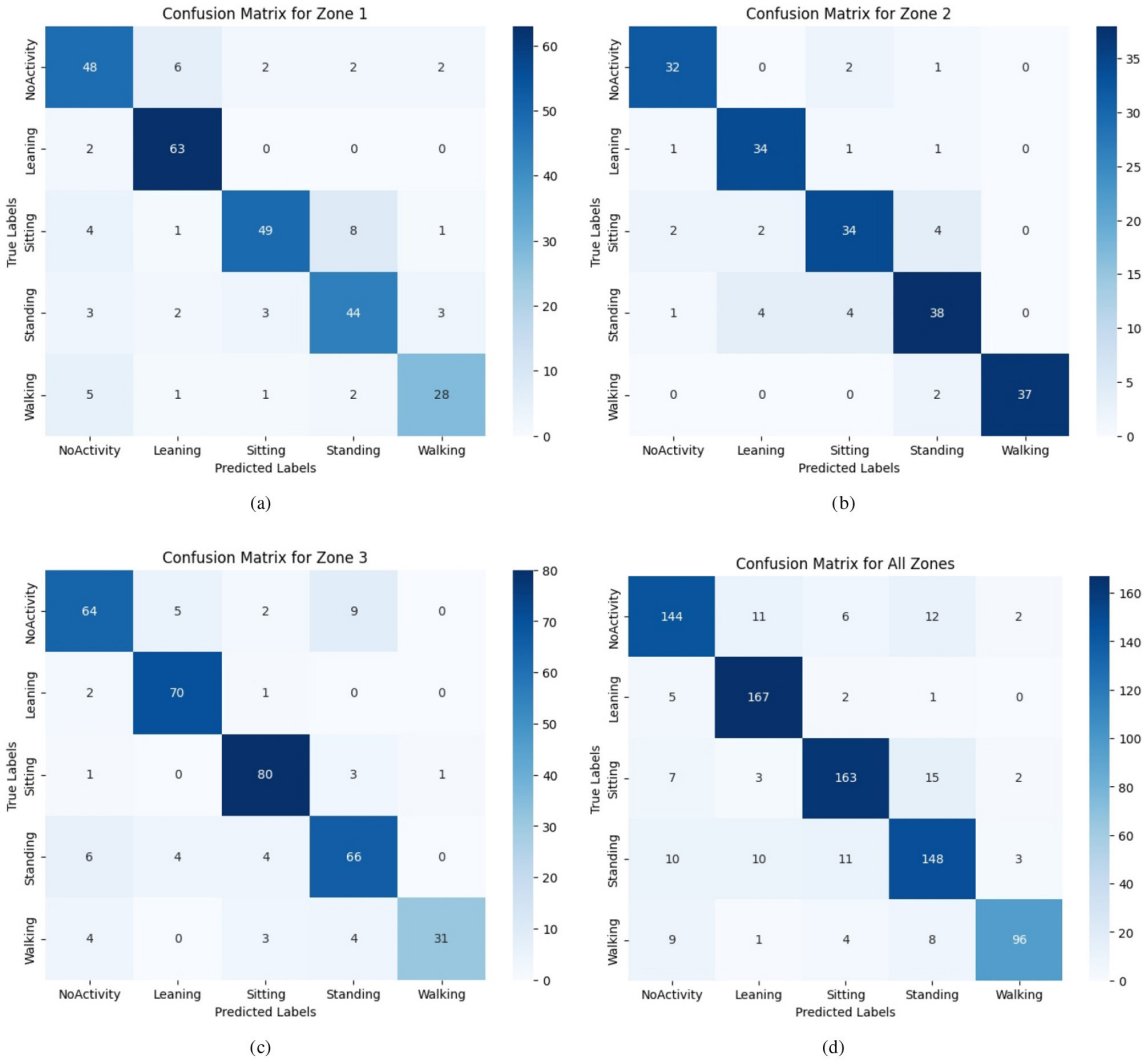
Confusion matrix for different zones. **(a)** Zone 1. **(b)** Zone 2. **(c)** Zone 3. **(d)** All zones.

In Figure 8, we present the classification report across different zones to evaluate the generalization of the global model across the local model's unseen datasets. As seen from Figure 8b, for Z2, walking activity is misclassified only two times, unlike Z1 and Z3, where it is more than 10, as the dataset across Z2 is homogeneous. This stark difference can be attributed to the homogeneity of the Z2 dataset, which enables the model to learn more accurate representations of the underlying classes. These results demonstrate the robustness of Fed-WAHAR in handling heterogeneous data while highlighting areas where further improvements can be made to address discrepancies across zones.

Fed-WAHAR is scalable as the aggregation is decentralized and parallelisable, making it feasible for expansion across hospitals or care homes. However, we acknowledge potential privacy limitations, including model inversion attacks, and recommend incorporating differential privacy or secure aggregation in future work to mitigate such risks.

## 6 Conclusion

This work addressed the challenge of location-centric CSI variability in HAR to detect daily activities such as standing, sitting, walking, and leaning with high precision in different zones. By integrating a dynamic weighting approach, Fed-WAHAR handles heterogeneous, Non-IID and location-sensitive data distributions generated across different zones to train a global FL model for improved cross-domain generalization. This research demonstrates the feasibility of effectively detecting human activities in collaborative settings, where the data generated by the entities exhibit heterogeneity and non-IID ness. Overall, the proposed Fed-WAHAR algorithm achieved a test accuracy of 85% across all zones, demonstrating its ability to generalize effectively in a federated learning setting. Future work will further enhance accuracy, extend the framework to additional cross-domain environments by varying SPECS, and incorporate advanced privacy-preserving mechanisms to improve data security in collaborative setups.

## Data Availability

Publicly available datasets were analyzed in this study. This data can be found here: Khan, M. Z., Taha, A., Taylor, W., Imran, M., and Abbasi, Q. (2022). *Non-invasive Localization using Software-Defined Radios*. https://researchdata.gla.ac.uk/1283/.

## References

[B1] Albogamy F. R. (2025). Federated learning for IOMT-enhanced human activity recognition with hybrid LSTM-GRU networks. Sensors 25:907. 10.3390/s25030907 39943546 PMC11820316

[B2] Antolinos E. Grajal J. (2025). “Improving vital signs monitoring in real-world environments with w-band phased-array radars,” in IEEE Transactions on Microwave Theory and Techniques. 10.1109/TMTT.2025.3544958

[B3] Bibbò L. Carotenuto R. Della Corte F. (2022). An overview of indoor localization system for human activity recognition (HAR) in healthcare. Sensors 22:8119. 10.3390/s22218119 36365817 PMC9656911

[B4] Chen Z. Cai C. Zheng T. Luo J. Xiong J. Wang X.. (2021). RF-based human activity recognition using signal adapted convolutional neural network. IEEE Trans. Mobile Comput. 22, 487–499. 10.1109/TMC.2021.3073969

[B5] Cheng D. Zhang L. Bu C. Wang X. Wu H. Song A.. (2023). Protohar: prototype guided personalized federated learning for human activity recognition. IEEE J. Biomed. Health Inform. 27, 3900–3911. 10.1109/JBHI.2023.3275438 37167056

[B6] Chopra N. Yang K. Abbasi Q. H. Qaraqe K. A. Philpott M. Alomainy A.. (2016). Thz time-domain spectroscopy of human skin tissue for in-body nanonetworks. IEEE Trans. Terahertz Sci. Technol. 6, 803–809. 10.1109/TTHZ.2016.2599075

[B7] Chourasia P. Ali T. E. Ali S. Pattersn M. (2024). DWFL: enhancing federated learning through dynamic weighted averaging. arXiv [Preprint]. arXiv:2411.05173. 10.48550/arXiv.2411.05173

[B8] Haider D. Ren A. Fan D. Zhao N. Yang X. Shah S. A.. (2019). An efficient monitoring of eclamptic seizures in wireless sensors networks. Comput. Electr. Eng. 75, 16–30. 10.1016/j.compeleceng.2019.02.011

[B9] Hernandez S. M. Bulut E. (2021). WiFederated: scalable WiFi sensing using edge-based federated learning. IEEE Internet Things J. 9, 12628–12640. 10.1109/JIOT.2021.3137793

[B10] Hong M. Kang S.-K. Lee J.-H. (2022). Weighted averaging federated learning based on example forgetting events in label imbalanced non-IID. Appl. Sci. 12:5806. 10.3390/app12125806

[B11] Khan I. Guerrieri A. Serra E. Spezzano G. (2025). A hybrid deep learning model for uwb radar-based human activity recognition. Internet Things 29:101458. 10.1016/j.iot.2024.101458

[B12] Khan M. Z. Taha A. Taylor W. Imran M. A. Abbasi Q. H. (2022). Non-invasive localization using software-defined radios. IEEE Sens. J. 22, 9018–9026. 10.1109/JSEN.2022.31607969874304

[B13] Klonovs J. Haque M. A. Krueger V. Nasrollahi K. Andersen-Ranberg K. Moeslund T. B.. (2015). Distributed Computing and Monitoring Technologies for Older Patients. Cham: Springer. 10.1007/978-3-319-27024-132593525

[B14] Kouhalvandi L. Karamzadeh S. (2025). Advances in non-contact human vital sign detection: a detailed survey of radar and wireless solutions. IEEE Access 13, 27833–27851. 10.1109/ACCESS.2025.3540716

[B15] Lévi-Strauss J. Marois C. Worbe Y. Bedoucha L. Benchikh Lehocine R. Rohaut B.. (2025). Utility and value of movement recording with combined EEG-EMG monitoring in the intensive care unit. Neurocrit. Care 1–12. 10.1007/s12028-025-02230-3 40032771

[B16] Li M. Qu L. Zhao Q. Guo J. Su X. Li X.. (2014). Precise point positioning with the BeiDou navigation satellite system. Sensors 14, 927–943. 10.3390/s140100927 24406856 PMC3926594

[B17] Li T. Sahu A. K. Zaheer M. Sanjabi M. Talwalkar A. Smith V.. (2020). Federated optimization in heterogeneous networks. Proc. Mach. Learn. Syst. 2, 429–450.

[B18] Li X. Song F. Luo M. Li K. Chang L. Chen X.. (2023). Towards collaborative and cross-domain Wi-Fi sensing: a case study for human activity recognition. IEEE Trans. Mobile Comput. 23, 1674–1688. 10.1109/TMC.2023.3242324

[B19] Liu J. Wang L. Guo L. Fang J. Lu B. Zhou W.. (2017). “A research on csi-based human motion detection in complex scenarios,” in 2017 IEEE 19th International Conference on e-Health Networking, Applications and Services (Healthcom) (Dalian: IEEE), 1–6. 10.1109/HealthCom.2017.8210800

[B20] López-Delgado I. E. Wang D. Fioranelli F. Grajal J. (2025). “Gait symmetry analysis with FMCW MIMO radar,” in IEEE Transactions on Microwave Theory and Techniques. 10.1109/TMTT.2025.3542183

[B21] Ma Z. Zhao M. Cai X. Jia Z. (2021). Fast-convergent federated learning with class-weighted aggregation. J. Syst. Architect. 117:102125. 10.1016/j.sysarc.2021.102125

[B22] Mendoza-Silva G. M. Torres-Sospedra J. Huerta J. (2019). A meta-review of indoor positioning systems. Sensors 19:4507. 10.3390/s19204507 31627331 PMC6832486

[B23] Naja S. Makhlouf M. Chehab M. A. H. (2017). An ageing world of the 21st century: a literature review. Int. J. Community Med. Public Health 4, 4363–4369. 10.18203/2394-6040.ijcmph20175306

[B24] Ouyang X. Xie Z. Zhou J. Xing G. Huang J. (2022). Clusterfl: a clustering-based federated learning system for human activity recognition. ACM Trans. Sens. Netw. 19, 1–32. 10.1145/3554980

[B25] Saeed U. Shah S. A. Khan M. Z. Alotaibi A. A. Althobaiti T. Ramzan N.. (2023). Software-defined radio-based contactless localization for diverse human activity recognition. IEEE Sens. J. 23, 12041–12048. 10.1109/JSEN.2023.3265867

[B26] Shi S. Sigg S. Chen L. Ji Y. (2018). Accurate location tracking from CSI-based passive device-free probabilistic fingerprinting. IEEE Tran. Veh. Technol. 67, 5217–5230. 10.1109/TVT.2018.2810307

[B27] Sophia S. Shankar B. M. Akshya K. Arunachalam A. C. Avanthika V. Deepak S.. (2021). “Bluetooth low energy based indoor positioning system using esp32,” in 2021 Third International Conference on Inventive Research in Computing Applications (ICIRCA) (Coimbatore: IEEE), 1698–1702. 10.1109/ICIRCA51532.2021.9544975

[B28] Sozinov K. Vlassov V. Girdzijauskas S. (2018). “Human activity recognition using federated learning,” in 2018 IEEE Intl Conf on Parallel &Distributed Processing with Applications, Ubiquitous Computing &Communications, Big Data &Cloud Computing, Social Computing &Networking, Sustainable Computing &Communications (ISPA/IUCC/BDCloud/SocialCom/SustainCom) (Melbourne, VIC: IEEE), 1103–1111. 10.1109/BDCloud.2018.00164

[B29] Stisen A. Blunck H. Bhattacharya S. Prentow T. S. Kjærgaard M. B. Dey A.. (2015). “Smart devices are different: assessing and mitigatingmobile sensing heterogeneities for activity recognition,” in Proceedings of the 13th ACM Conference on Embedded Networked Sensor Systems (New York, NY: ACM), 127–140. 10.1145/2809695.2809718

[B30] Taylor W. Shah S. A. Dashtipour K. Zahid A. Abbasi Q. H. Imran M. A.. (2020). An intelligent non-invasive real-time human activity recognition system for next-generation healthcare. Sensors 20:2653. 10.3390/s20092653 32384716 PMC7248832

[B31] Wang X. Yang C. Mao S. (2020). On CSI-based vital sign monitoring using commodity wifi. ACM Trans. Comput. Healthc. 1, 1–27. 10.1145/337716539598974

[B32] Wen X. Wang Y. Yuan M. Geng Y. Yu H. Zheng G.. (2024). Enhancing human activity recognition with FEDPA: focusing on non-IID data challenges in federated learning. IEEE Sens. J. 10.1109/JSEN.2024.3465593

[B33] Xiao Z. Xu X. Xing H. Song F. Wang X. Zhao B.. (2021). A federated learning system with enhanced feature extraction for human activity recognition. Knowl.-Based Syst. 229:107338. 10.1016/j.knosys.2021.10733839943546

[B34] Yang Z. Wu C. Zhou Z. Zhang X. Wang X. Liu Y.. (2015). Mobility increases localizability: a survey on wireless indoor localization using inertial sensors. ACM Comput. Surv. 47, 1–34. 10.1145/2676430

[B35] Yang Z. Zhou Z. Liu Y. (2013). From RSSI to CSI: indoor localization via channel response. ACM Comput. Surv. 46, 1–32. 10.1145/2543581.2543592

[B36] Yurtman A. Barshan B. (2016). Human activity recognition using tag-based radio frequency localization. Appl. Artif. Intell. 30, 153–179. 10.1080/08839514.2016.1138787

[B37] Zhang S. Jia H. Jiang T. Wu S. Ding X. Zhong Y.. (2025). Federated learning framework for Wi-Fi-based cross-environment human action recognition. Measurement 253:117821. 10.1016/j.measurement.2025.117821

